# Spatial genomic, biochemical, and cellular mechanisms drive meningioma heterogeneity and evolution

**DOI:** 10.21203/rs.3.rs-2921804/v1

**Published:** 2023-05-15

**Authors:** Calixto-Hope Lucas, Kanish Mirchia, Kyounghee Seo, Hinda Najem, William Chen, Naomi Zakimi, Abrar Choudhury, S. John Liu, Joanna Phillips, Stephen Magill, Craig Horbinski, David Solomon, Arie Perry, Harish Vasudevan, Amy Heimberger, David Raleigh

**Affiliations:** Johns Hopkins University; Univeristy of California San Francisco; Univeristy of California San Francisco; Northwestern University; UCSF; Univeristy of California San Francisco; University of California, San Francisco; University of California San Francisco; UCSF; Northwestern University; Northwestern University; University of California, San Francisco; UCSF; University of California San Francisco; Northwestern University; University of California San Francisco

## Abstract

Intratumor heterogeneity underlies cancer evolution and treatment resistance^1–5^, but targetable mechanisms driving intratumor heterogeneity are poorly understood. Meningiomas are the most common primary intracranial tumors and are resistant to all current medical therapies^6,7^. High-grade meningiomas cause significant neurological morbidity and mortality and are distinguished from low-grade meningiomas by increased intratumor heterogeneity arising from clonal evolution and divergence^8^. Here we integrate spatial transcriptomic and spatial protein profiling approaches across high-grade meningiomas to identify genomic, biochemical, and cellular mechanisms linking intratumor heterogeneity to the molecular, temporal, and spatial evolution of cancer. We show divergent intratumor gene and protein expression programs distinguish high-grade meningiomas that are otherwise grouped together by current clinical classification systems. Analyses of matched pairs of primary and recurrent meningiomas reveal spatial expansion of sub-clonal copy number variants underlies treatment resistance. Multiplexed sequential immunofluorescence (seqIF) and spatial deconvolution of meningioma single-cell RNA sequencing show decreased immune infiltration, decreased MAPK signaling, increased PI3K-AKT signaling, and increased cell proliferation drive meningioma recurrence. To translate these findings to clinical practice, we use epigenetic editing and lineage tracing approaches in meningioma organoid models to identify new molecular therapy combinations that target intratumor heterogeneity and block tumor growth. Our results establish a foundation for personalized medical therapy to treat patients with high-grade meningiomas and provide a framework for understanding therapeutic vulnerabilities driving intratumor heterogeneity and tumor evolution.

Meningiomas arising from the meningothelial lining of the central nervous system comprise more than 40% of primary intracranial tumors^7,9^, and approximately 1% of humans will develop a meningioma in their lifetime^10^. Bioinformatic investigations have shed light on mechanisms underlying meningioma tumorigenesis^11–18^, but current meningioma classification systems are based on histological and/or molecular approaches that can be confounded by intratumor heterogeneity^8,19^. High-grade meningiomas are particularly heterogeneous^8,20^ and are prone to recurrence and decreased survival despite treatment with surgery and radiotherapy^21^. Medical therapies remain ineffective or experimental for meningiomas^6^, and intratumor heterogeneity and tumor evolution in response to treatment have undermined all clinical trials for patients with meningiomas^22,23^. Glioblastoma evolution also selects for treatment resistant sub-clones^1,5^, and sub-clonal somatic short variants (SSVs) or copy number variants (CNVs) are associated with non-small cell lung cancer recurrence, the leading cause of cancer-related death worldwide^4^. Evolving sub-clones underlie increased mortality across many human cancers^2^, and while genetic drivers of the initial stages of tumorigenesis are well described^3^, the identity of mechanisms driving intratumor heterogeneity and tumor evolution are incompletely understood.

Here we test the hypothesis that understanding spatial genomic, biochemical, and cellular mechanisms linking intratumor heterogeneity to tumor evolution may reveal druggable dependencies underlying human cancer. To do so, we use spatial transcriptomic and protein profiling approaches to study clinical samples from high-grade human meningiomas (Fig. 1). When integrated with clinically established histological or bulk molecular approaches for meningioma classification^13,20,24^, multiplexed sequential immunofluorescence (seqIF), and spatial deconvolution of meningioma single-cell RNA sequencing, our results elucidate how intratumor heterogeneity influences the molecular (Fig. 2), temporal (Fig. 3, 4), and spatial evolution (Fig. 5, 6) of the most common primary intracranial tumor^7,9^. To validate these findings and generate a platform for testing personalized medical therapies to treat high-grade meningiomas, we use CRISPR interference (CRISPRi)^25^ and fluorescent labeling of human meningioma cells in preclinical organoid models to identify new combinations of FDA-approved small molecules that inhibit intratumor heterogeneity and block meningioma growth (Fig. 7).

## Experimental design and workflow

**Figure 1:**
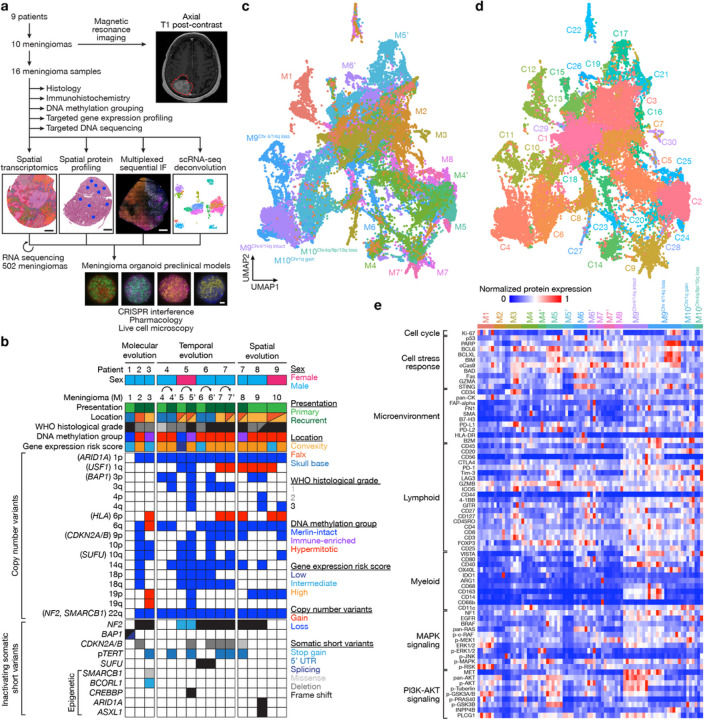
Experimental design and workflow. **a**, 16 high-grade meningioma samples from 10 meningiomas that were resected from 9 patients were analyzed using comprehensive histologic, immunohistochemical, and bulk and spatial bioinformatic techniques, including spatial transcriptomics, spatial protein profiling, multiplexed sequential immunofluorescence microscopy, and spatial deconvolution of meningioma single-cell RNA sequencing. Results were validated using RNA sequencing from 502 meningiomas, and CRISPR interference, pharmacology, and live cell imaging of meningioma organoid preclinical models. Scale bars, 1mm for meningiomas and 100μm for meningioma organoids. **b**, Oncoprint comprised of the clinical, histologic, genetic, epigenetic, and gene expression features of the meningioma samples in this study. **c**, Uniform manifold approximation and projection (UMAP) of 38,718 meningioma spatial transcriptomes after Harmony batch correction shaded by sample of origin. **d**, UMAP of meningioma spatial transcriptomes after Harmony batch correction shaded by unsupervised hierarchical clusters. **e**, Heatmap of meningioma spatial protein profiling comprised of 72 proteins from 82 regions revealing significant inter- and intratumor heterogeneity.

To define mechanisms underlying meningioma intratumor heterogeneity and evolution, 16 intracranial samples from 10 meningiomas (designated M1–10) that were resected from 9 patients at the University of California San Francisco (UCSF) were assembled for clinical, histological, and molecular analyses (Fig. 1a and Supplementary Table 1). Preoperative magnetic resonance imaging (MRI) studies and medical records were reviewed to define meningioma locations, presentations (primary versus recurrent), treatments, and outcomes. Histological and molecular analyses of all samples were performed using the most recent criteria from the World Health Organization (WHO) Classification of Tumors of the Central Nervous System^20^, including targeted next generation DNA sequencing^26^ to define SSVs and CNVs that are associated with high-grade meningioma classification and adverse clinical outcomes^15,16, 27–29^ (Supplementary Tables 1 and 2). All samples were analyzed using immunohistochemistry (IHC) for cell proliferation (Ki-67), cell cycle regulation (p16), or chromatin markers (H3K27me^3^), each of which can also be associated with clinical outcomes for patients with meningiomas^30–32^. To integrate standard approaches for meningioma classification with emerging techniques that define biological drivers and therapeutic vulnerabilities in meningiomas, DNA methylation grouping^13^ and targeted gene expression profiling^24^ were performed on all samples (Supplementary Tables 1 and 3). These comprehensive clinical, histological, and molecular analyses identified meningiomas to study the molecular (M1–3), temporal (M4–7), and spatial evolution (M8–10) of human cancer (Fig. 1b).

Spatial transcriptomic profiling of 50μm regions from continuous arrays tiled across 6mm cores was performed on all meningiomas using an approach that integrates approximately 10 cells per capture area^33^ (Extended Data Fig. 1a). Core selection for each sample was guided by morphological or IHC heterogeneity of whole mount formalin-fixed paraffin-embedded (FFPE) tumor sections. Spatial transcriptomes were aligned with hematoxylin and eosin (H&E) histological images using unique oligonucleotide barcodes corresponding to array positions (Extended Data Fig. 1b). The Harmony bioinformatic pipeline was used for sample integration and batch-correction^34^ (Extended Data Fig. 1c), and uniform manifold approximation and projection (UMAP) analysis of 38,718 spatial transcriptomes demonstrated 30 spatial gene expression programs across 16 high-grade meningioma samples (range: 4–15 programs/sample) (Fig. 1c, d, Extended Data Fig. 2a–c and Supplementary Table 4). Six spatial gene expression programs that included transcriptomes from all samples were distinguished by enrichment of genes involved in neural development (*SIM2, VIT* in C1 and C7), angiogenesis (*THBS2, HHIP* in C3), meningeal homeostasis and neurotransmitter processing (*PTGDS, LCNL1* in C5), bone differentiation (*MAP1LC3C, ALPL* in C9), and differentiation of the neural crest (*S100A, S100B* in C14), a multipotent embryonic cell population that gives rise to the meninges^35,36^ (Extended Data Fig. 2a–c and Supplementary Table 4).

Spatial protein profiling of 200μm regions from 6mm cores was performed on all meningiomas using an approach that integrates approximately 190 cells per capture area^37^ (range 115–283 cells/region). Region selection for each sample was guided by morphological or IHC heterogeneity of whole mount FFPE tumor sections. Laser microdissection and next generation sequencing was used to quantify binding of 72 antibodies that were conjugated to unique oligonucleotide barcodes from 82 regions (range: 3–12 regions/sample) (Fig. 1e and Supplementary Table 5). Principal component analysis of spatial protein profiling data demonstrated divergent biochemical mechanisms within and across high-grade meningiomas (Extended Data Fig. 2d).

**Figure 2:**
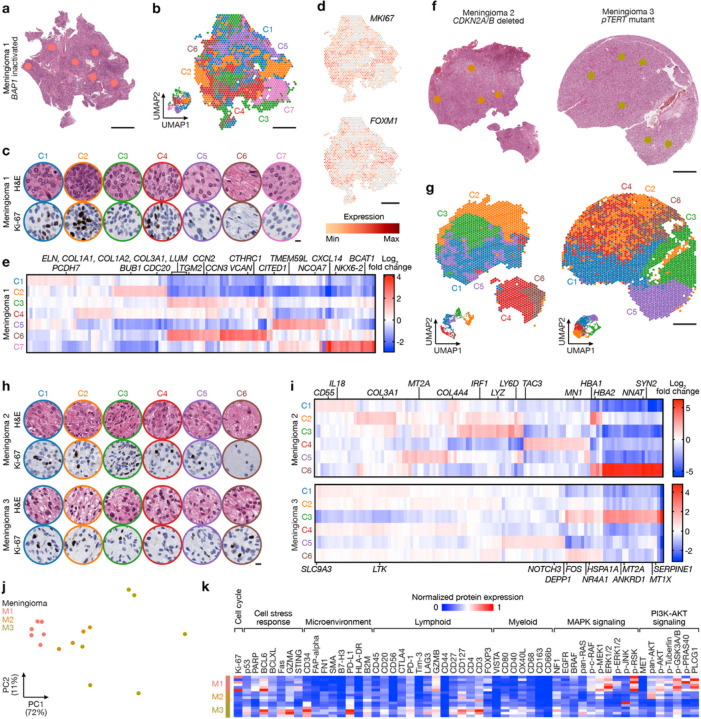
High-grade meningiomas are distinguished by divergent intratumor gene and protein expression programs. Spatial transcriptomics and protein profiling of meningiomas 1–3 (M1–3) with driver mutations associated with adverse clinical outcomes, such as *BAP1* loss (M1), *CDKN2A/B* loss (M2), or *TERT* promoter mutation (M3). **a**, M1 H&E-stained section showing regions of spatial protein profiling. Scale bar, 1mm. **b**, Spatial distribution of unsupervised hierarchical spatial transcriptome clusters from M1. Insert shows Uniform manifold approximation and project (UMAP) analysis of M1 spatial transcriptomes. Scale bar, 1mm. **c**, Representative H&E morphology and Ki-67 immunohistochemistry (IHC) of spatial transcriptome clusters from M1. Colors correspond to spatial transcriptomes from **b**. Scale bar, 10μm. **d**, Spatial distribution and expression of *MKI67* or *FOXM1* transcripts from M1. Scale bar, 1mm. **e**, Top 119 differentially expressed genes across unsupervised hierarchical spatial transcriptome clusters from M1. **f**, M2 (left) or M3 (right) H&E-stained sections showing regions of spatial protein profiling. Scale bar, 1mm. **g**, Spatial distribution of unsupervised hierarchical spatial transcriptome clusters from M2 (left) or M3 (right). Inserts show UMAP analyses of M2 or M3 spatial transcriptomes. Scale bar, 1mm. **h**, Representative H&E morphology and Ki-67 IHC of spatial transcriptome clusters from M2 (top) or M3 (bottom). Colors correspond to spatial transcriptomes from **g**. Scale bar, 10μm. **i**, Top differentially expressed genes across unsupervised hierarchical spatial transcriptome clusters from M2 (top, 115 genes) or M3 (bottom, 110 genes). **j**, Principal component (PC) analysis of spatial protein profiling from M1–3. **k**, Differentially expressed spatial proteins from M1–3 (all with Student’s t test p£0.05 for head-to-head comparisons of one meningioma to at least one other meningioma).

**Figure 3:**
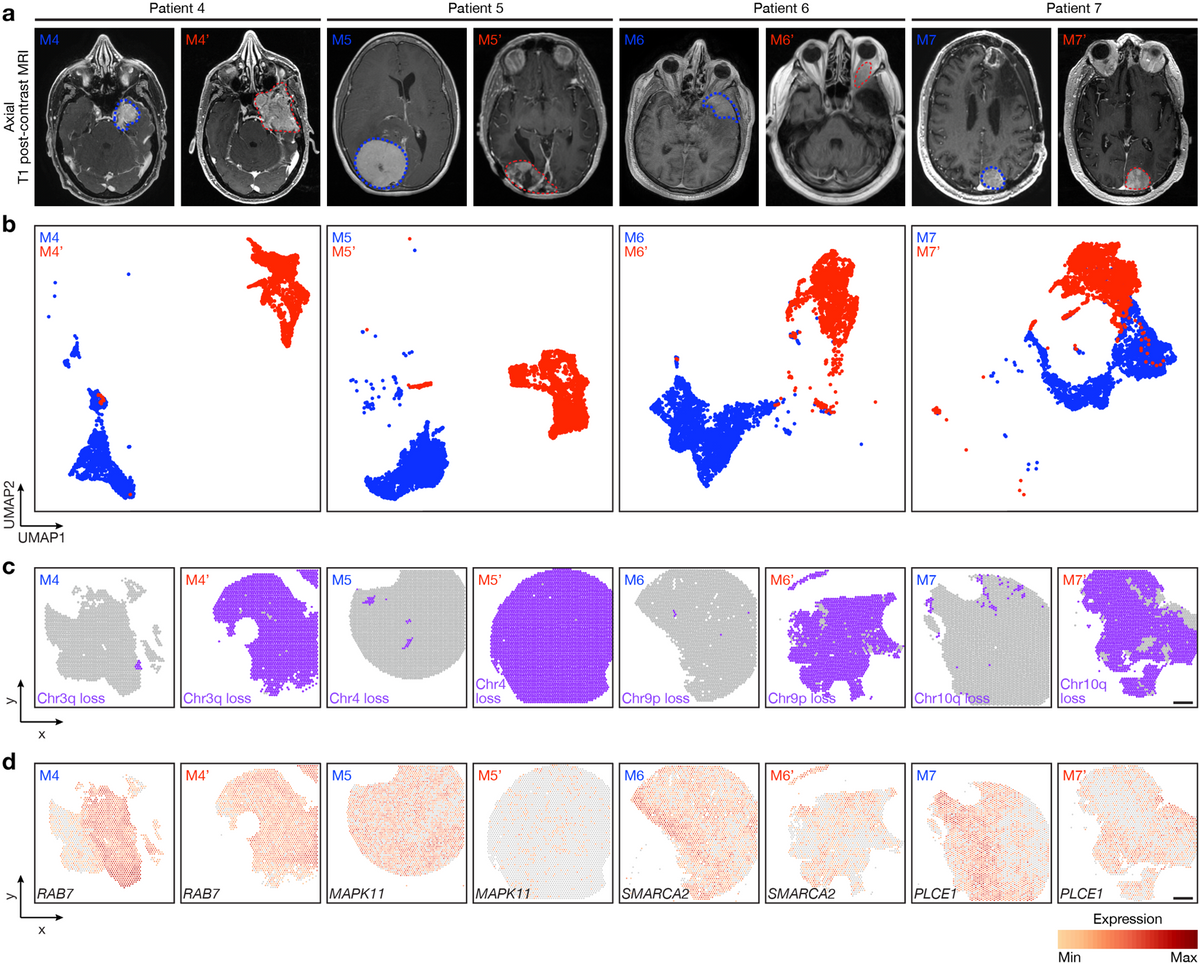
Spatial expansion of sub-clonal copy number variants underlies high-grade meningioma recurrence. Spatial transcriptomics and protein profiling of matched pairs of primary and recurrent meningiomas from patients 4–7 (M4 and M4’, M5 and M5’, M6 and M6’, and M7 and M7’). **a**, Preoperative T1 post-contrast magnetic resonance imaging (MRI) of matched pairs of primary (blue, M4, M5, M6, M7) and recurrent (red, M4’, M5’, M6’, M7’) meningiomas. **b**, UMAP analysis of matched pairs of primary and recurrent meningioma spatial transcriptomes after Harmony batch correction. Scale bar, 1mm. **c**, Spatial distribution of unsupervised hierarchical spatial transcriptome clusters harboring divergent copy number variants from InferCNV. Scale bar, 1mm. **d**, Spatial distribution of differentially expressed genes associated with copy number variants across matched pairs of primary and recurrent meningiomas. Scale bar, 1mm.

**Figure 4:**
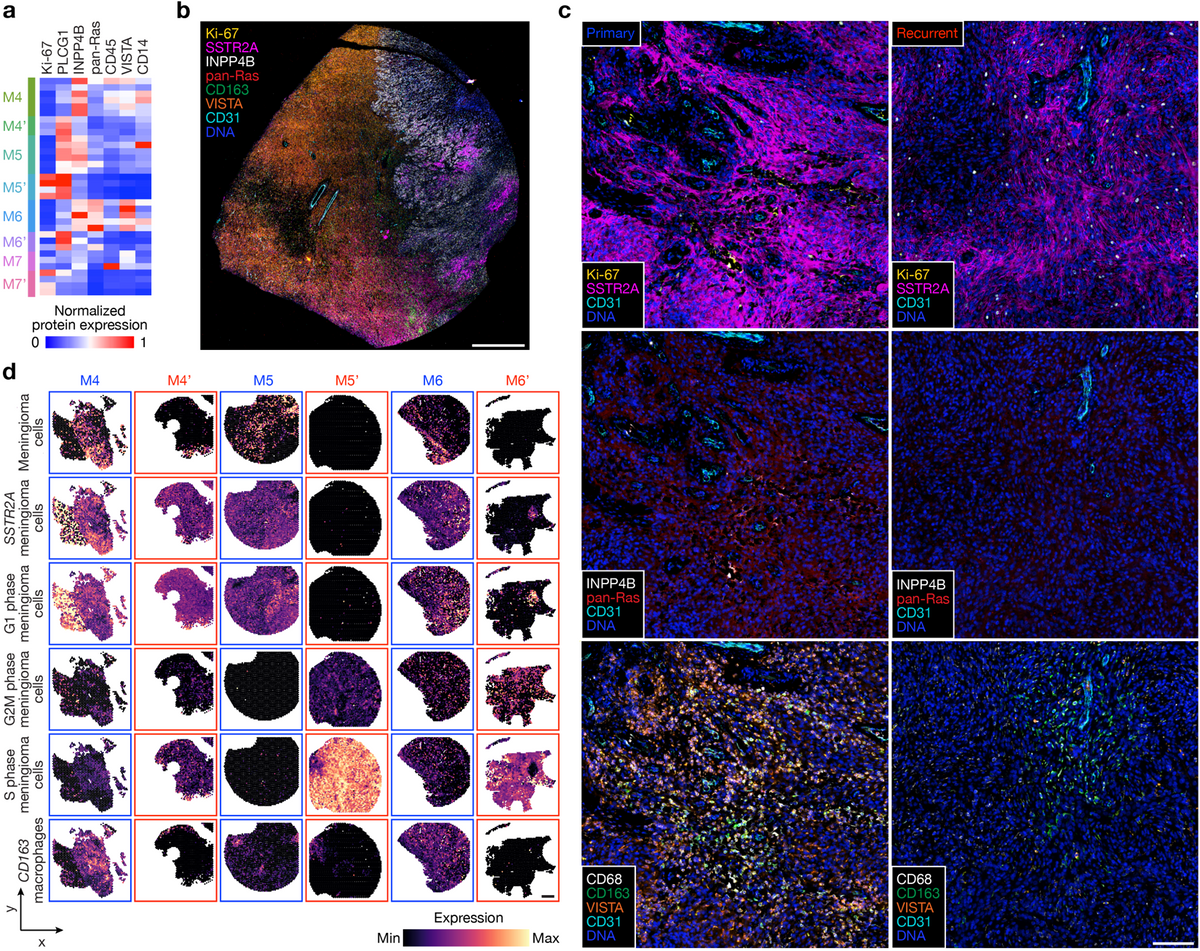
Decreased immune infiltration, decreased MAPK signaling, increased PI3K-AKT signaling, and increased cell proliferation underlie high-grade meningioma recurrence. **a**, Differentially expressed spatial proteins from M4–7’ (all with Student’s t test p£0.05 for at least 3 of 4 primary versus recurrent meningioma comparisons). **b**, Representative image of multiplexed seqIF microscopy showing intratumor heterogeneity of signaling mechanisms and cell types in the region of M9 with WHO grade 2 (left) and WHO grade 3 (right) histology, as well as *ARID1A* and Chr4/14q loss. Scale bar, 1mm. **c**, Multiplexed seqIF microscopy showing temporal evolution of signaling mechanisms and cell types in primary versus recurrent meningiomas. Images from M4 and M4’ that are representative of matched pairs of primary and recurrent meningiomas from patients 4–7 (M4 and M4’, M5 and M5’, M6 and M6’, and M7 and M7’). Scale bar, 100μm. **d**, Spatial deconvolution of meningioma single-cell RNA sequencing showing temporal evolution of cell types from matched pairs of primary (blue) and recurrent (red) meningiomas. Scale bar, 1mm.

**Figure 5:**
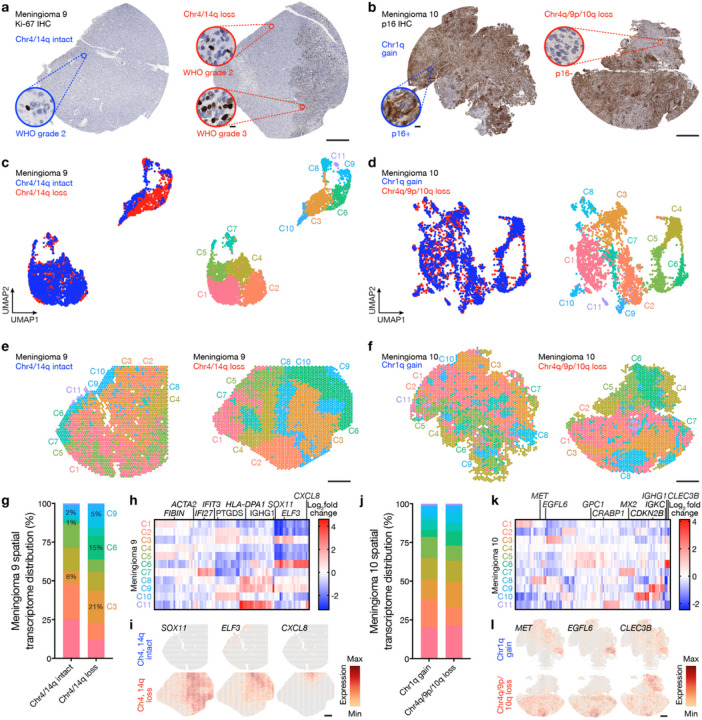
Regionally distinct sub-clonal spatial transcriptomes underlie histological heterogeneity in high-grade meningioma. **a**, Ki-67 immunohistochemistry (IHC) of regionally distinct samples from M9 demonstrating heterogeneous histological (WHO grade 2 or 3), mutational (*ARID1A, ASXL1*), and cytogenetic (chromosome 4, 14q) features (Fig. 1b). **b**, p16 IHC of regionally distinct samples from M10 demonstrating heterogeneous histological (p16, Ki-67) and cytogenetic (chromosome 1q, 4q, 9p, 10q) features (Fig. 1b). **c**, UMAP analysis of M9 spatial transcriptomes after Harmony batch correction shaded by region of origin (left) or unsupervised hierarchical clusters (right). Scale bar, 1mm. **d**, UMAP analysis of M10 spatial transcriptomes after Harmony batch correction shaded by region of origin (left) or unsupervised hierarchical clusters (right). Scale bar, 1mm. **e**, Spatial distribution of unsupervised hierarchical spatial transcriptome clusters from M9 after Harmony batch correction. Scale bar, 1mm. **f**, Spatial distribution of unsupervised hierarchical spatial transcriptome clusters from M10 after Harmony batch correction. Scale bar, 1mm. **g**, Distribution of unsupervised hierarchical spatial transcriptome clusters from M9 after Harmony batch correction. Spatial transcriptome clusters correlating with WHO grade 3 histology are annotated. **h**, Top 89 differentially expressed genes across unsupervised hierarchical spatial transcriptome clusters from M9. **I**, Spatial distribution of differentially expressed genes associated with histological variability across regionally distinct samples from M9. Scale bar, 1mm. **j**, Distribution of unsupervised hierarchical spatial transcriptome clusters from M10 after Harmony batch correction. **k**, Top 110 differentially expressed genes across unsupervised hierarchical spatial transcriptome clusters from M10. **l**, Spatial distribution of differentially expressed genes associated with histological variability across regionally distinct samples from M10. Scale bar, 1mm.

**Figure 6:**
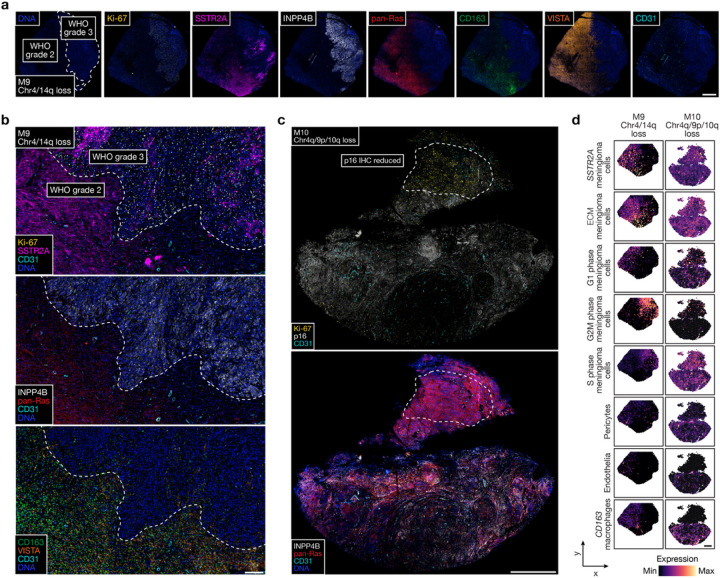
High-grade meningiomas are distinguished by regionally distinct intratumor immune infiltration, MAPK signaling, PI3K-AKT signaling, and cell proliferation. **a**, Multiplexed seqIF microscopy showing intratumor heterogeneity of signaling mechanisms and cell types in the region of M9 with WHO grade 2 (left) and WHO grade 3 (right) histology, as well as *ARID1A* and Chr4/14q loss. Scale bar, 1mm. **b**, Multiplexed seqIF microscopy showing M9 from **a** at higher magnification. Scale bar, 200μmm. **c**, Multiplexed seqIF microscopy showing intratumor heterogeneity of signaling mechanisms in the region of M10 with reduced immunostaining for p16 (top) and Chr4q/9p/10q loss. Scale bar, 1mm. **d**, Spatial deconvolution of meningioma single-cell RNA sequencing showing spatial evolution of cell types from in M9 in **a** and **b** (left) or M10 in **c** (right). Scale bar, 1mm.

**Figure 7:**
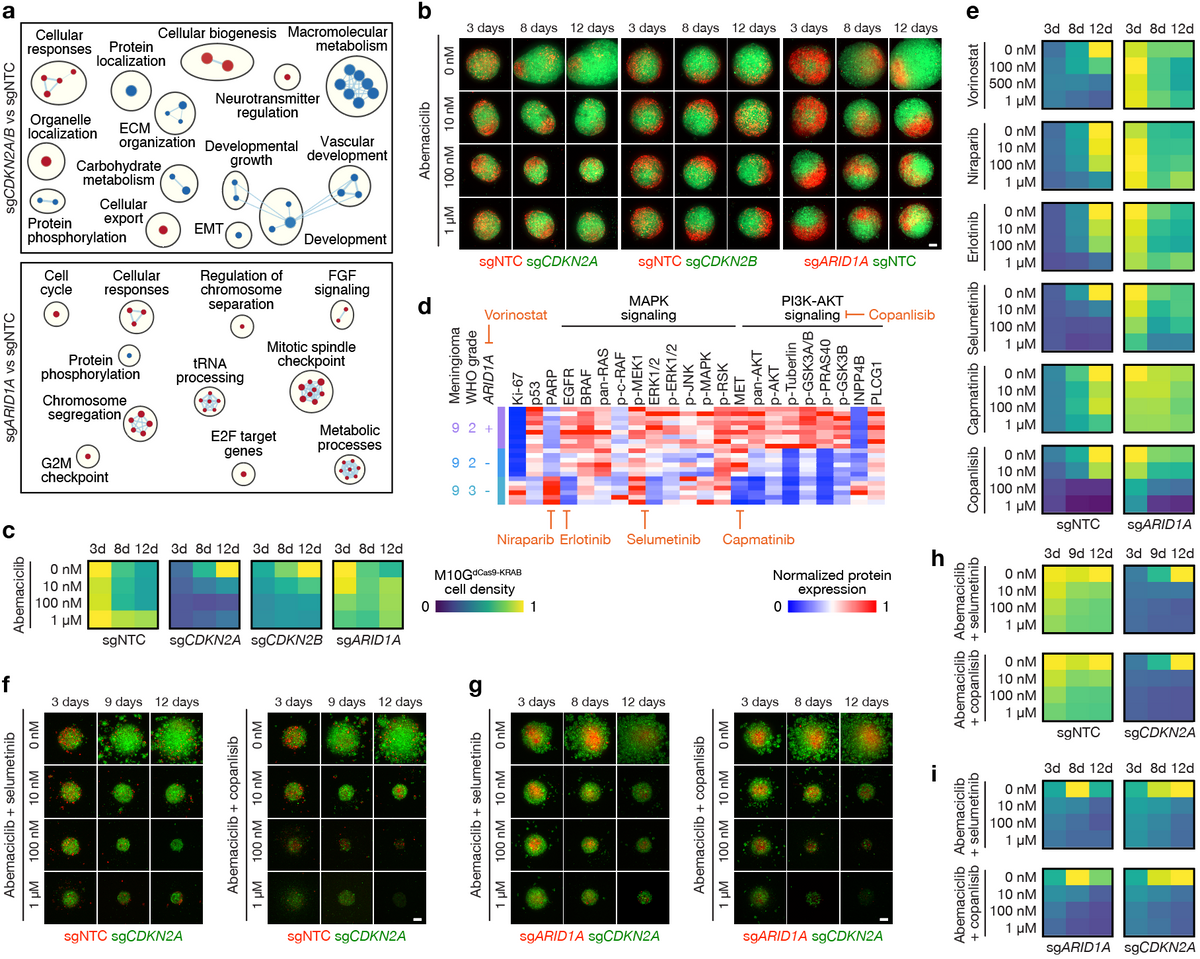
A preclinical platform for testing personalized medical therapies to overcome intratumor heterogeneity in high-grade meningiomas. **a**, Network of gene circuits distinguishing M10G^dCas9-KRAB^ meningioma cells expressing sgNTC (n=3), sg*CDKN2A* (n=3), sg*CDKN2B* (n=3), or sg*ARID1A* (n=3) using RNA sequencing. Nodes represent pathways and edges represent shared genes between pathways (p£0.05, FDR£0.05). Red nodes are enriched and blue nodes are suppressed in experimental versus sgNTC control conditions. **b**, Abemaciclib treatments of 3D organoid co-cultures of M10G^dCas9-KRAB^ meningioma cells expressing sgNTC, sg*CDKN2A*, sg*CDKN2B*, or sg*ARID1A*. Scale bar, 100μm. **c**, Quantification of abemaciclib treatments of 3D organoid co-cultures of M10G^dCas9-KRAB^ meningioma cells expressing sgNTC, sg*CDKN2A*, sg*CDKN2B*, or sg*ARID1A*. Representative of 8–10 biological replicates per condition. **d**, Differentially expressed spatial proteins from M9 (all with Student’s t test p£0.05 for at least 2 of 3 regionally distinct comparisons). **e**, Quantification of molecular therapy treatments of 3D organoid co-cultures of M10G^dCas9-KRAB^ meningioma cells expressing sgNTC or sg*ARID1A*. Representative of 8–10 biological replicates per condition. Scale from **c. f**, Combination molecular therapy treatments of 3D organoid co-cultures of M10G^dCas9-KRAB^ meningioma cells expressing sg*CDKN2A* or sgNTC. Scale bar, 100μm. **g**, Combination molecular therapy treatments of 3D organoid co-cultures of M10G^dCas9-KRAB^ meningioma cells expressing sg*CDKN2A* or sg*ARID1A*. Scale bar, 100μm. **h**, Quantification of combination molecular therapy treatments of 3D organoid co-cultures of M10G^dCas9-KRAB^ meningioma cells expressing sg*CDKN2A* or sgNTC. Representative of 8 biological replicates per condition. Scale from **c. i**, Quantification of combination molecular therapy treatments of 3D organoid co-cultures of M10G^dCas9-KRAB^ meningioma cells expressing sg*CDKN2A* or sg*ARID1A*. Representative of 8 biological replicates per condition. Scale from **c**.

Using these clinical, histological, molecular, and spatial data, the study cohort was divided into 3 groups to study genomic, biochemical, and cellular mechanisms underlying intratumor heterogeneity in the context of molecular (Fig. 2), temporal (Fig. 3, 4), and spatial evolution of meningiomas (Fig. 5, 6). Findings were validated using multiplexed seqIF microscopy, spatial deconvolution of meningioma single-cell RNA sequencing, bulk RNA sequencing from 502 meningiomas^13,18^, and CRISPR interference, pharmacology, and live cell imaging in meningioma organoid preclinical models (Fig. 7).

## High-grade meningiomas are distinguished by divergent intratumor gene and protein expression programs

The WHO defines meningioma grades according to histological features, such as mitotic count and brain invasion, and rare molecular features such as *CDKN2A/B* homozygous deletions or hotspot *TERT* promoter mutations that are sufficient for diagnosis of WHO grade 3 meningioma^20^. Most WHO grade 1 meningiomas can be effectively treated with surgery or radiotherapy, but many WHO grade 2 or grade 3 (high-grade) meningiomas, which account for 20–30% of cases^7,9^, are resistant to treatment and cause significant neurological morbidity and mortality^6^. Morphological features can influence meningioma WHO grading, and rhabdoid morphology associated with inactivating *BAP1* mutation is also associated with WHO grade 3 meningioma^38^. Thus, current clinical classification systems group meningiomas with different driver mutations into the same high-grade group, which may not provide an optimal framework for clinical trials. To determine if high-grade meningiomas were associated with convergent or divergent intratumor gene or protein expression programs, spatial genomic and biochemical mechanisms were studied across meningiomas with *BAP1* inactivation (M1), *CDKN2A/B* homozygous deletion (M2), or *TERT* promoter mutation (M3) (Extended Data Fig. 3a, b and Supplementary Table 1).

Spatial gene expression programs in M1 correlated with morphological features and immunostaining for Ki-67 (Fig. 2a–c). Spatial transcriptomes with increased immunostaining for Ki-67 (C2, C4) were enriched in *MKI67* and *FOXM1*, a driver of meningioma cell proliferation that is enriched in high-grade meningiomas or meningiomas from the Hypermitotic DNA methylation group^13,39^ (Fig. 2d). Differential expression analysis of spatial transcriptomes identified 2 clusters (C3, C6) that were distinguished by expression of collagens (*COL1A1, COL1A2, COL3A1*) and other extracellular matrix genes (*LUM, ELN, VCAN*) and correlated with regions of increased connective tissue on H&E sections (Fig. 2a, c, e and Extended Data Fig. 3b). The remaining spatial gene expression programs in M1 were comprised of variably cellular tumor with differential expression of Wnt pathway (*CTHRC1, TMEM59L*), inflammatory (*CXCL14*), cell proliferation (*CCN2, CCN3, CITED1, BCAT1, NCOA7*), cell differentiation (*NKX6–2*), or cell adhesion genes (*PCDH7, TGM2*) (Fig. 2e). Clusters with increased immunostaining for Ki-67 were distinguished by non-overlapping cell proliferation genes (*BUB1*, *CDC20* in C2, *CCN3* in C4) (Fig. 2d, e), suggesting regionally distinct mechanisms activating the cell cycle can exist in individual tumors.

Spatial gene expression programs in M2 and M3 also demonstrated heterogeneous ontologies that correlated with morphological features (Fig. 2f–I and Extended Data Fig. 3b). Differential expression analysis of spatial transcriptomes in M2 revealed a connective tissue and hemorrhagic cluster (*COL3A1*, *COL4A4, HBA1, HBA2* in C2), a brain parenchyma cluster (*NNAT, SYN2* in C6), and 4 other clusters comprised of variably cellular tumor that were distinguished by enrichment of inflammatory and immune genes (*IRF1, CD55, IL18*, *LYZ*, *LY6D*) (Fig. 2f, h, I and Extended Data Fig. 3b). C4 was comprised of brain-invasive meningioma with enrichment of oncogenes (*MN1*) and tissue invasion genes (*TAC3*). C5 from M2 and C3 from M3 showed decreased expression of inflammatory and immune genes but enrichment of *MT2A*, which is implicated in cell stress, homeostasis, and differentiation^40,41^. Other cell stress genes and DNA damage response genes were enriched in C3 from M3 (*HSP1A, NR4A1, ANKRD1*), and the 5 other spatial gene expression programs in M3 were distinguished by differential expression of ion transport, cell stress, and immune genes that were not differentially expressed in M1 or M2 (*SLC9A3*, *LTK*, *DEPP1*, *HSP1A*, *NOTCH3, FOS, SERPINE1, MT1X*) (Fig. 2i).

Spatial protein profiling validated divergent signaling mechanisms across spatial transcriptomes from high-grade meningiomas, and revealed heterogeneous cell proliferation, stress, microenvironment, immune, and growth factor signaling pathways across M1–3 (Fig. 2i–j). Sample integration and batch-correction with Harmony was unable to identify conserved spatial gene expression programs across M1–3 (Extended Data Fig. 3c–e). In support of these data, differential expression analyses using bulk RNA sequencing data from independent meningiomas with *BAP1* inactivation (n = 5), *CDKN2A/B* homozygous deletion (n = 30), or *TERT* promoter mutation (n = 7) that were resected at UCSF or The University of Hong Kong (HKU)^13,18^ showed differences in diverse cell proliferation (*MKI67, FOXM1, CCN2, CCN3, CITED1, BCAT1, NCOA7, BUB1, CDC20*), differentiation (*CTHRC1, MT2A, NKX6–2*), tissue invasion and adhesion (*LUM, TAC3, PCDH7, TGM2*), immune (*CXCL14, IL18, LYZ, LY6D*), and tumor suppressor genes (*WT1, MN1*) (Supplementary Table 6). Thus, high-grade meningiomas with different driver mutations that are grouped together by current clinical classification systems are distinguished by divergent intratumor gene and protein expression programs. These data suggest that unsuccessful remote^22^ or recent^23^ clinical trials of molecular therapy for patients with meningiomas may have benefitted from molecular rather than histological criteria for inclusion and treatment.

## Spatial sub-clonal copy number variants, signaling mechanisms, cell types underlie high-grade and meningioma recurrence

Surgery is the mainstay of meningioma treatment, but postoperative radiotherapy is recommended to reduce the risk of high-grade meningioma recurrence^9,42^. Nevertheless, local recurrence of high-grade meningioma is common^21^, and recurrence is the leading cause of death in patients with meningiomas that are resistant to standard interventions^43^. Mechanisms underlying meningioma resistance to treatment are poorly understood. To address this limitation in our understanding of meningioma biology, spatial genomic, biochemical, and cellular mechanisms were studied in the context of histological and molecular classification systems across matched pairs of primary (M4–7) and recurrent (M4’–7’) high-grade meningiomas that were treated with radiotherapy between primary and recurrent resections (Fig. 3a, Extended Data Fig. 4a and Supplementary Table 1).

Histological analysis showed higher WHO grades and increased immunostaining for Ki-67 in paired recurrent versus primary meningiomas (Fig. 1b and Extended Data Fig. 4a). Bulk molecular approaches demonstrated higher gene expression risk scores, increased CNV burden, and aggressive driver mutations such as *TERT* promoter mutation or *CDKN2A/B* homozygous deletion in paired recurrent versus primary meningiomas (Fig. 1b and Supplementary Table 2, 3). Spatial gene expression programs were divergent in paired primary and recurrent meningiomas despite sample integration and batch-correction with Harmony (Fig. 3b and Extended Data Fig. 4b–d). Incorporation of CNVs can improve prognostic models for meningioma outcomes^15,16^, but the spatial architecture and evolution of meningioma CNVs over time is incompletely understood. To determine if spatial expansion of sub-clonal CNVs underlies high-grade meningioma recurrence, inferCNV^44,45^ was used to deconvolve paired primary and recurrent meningioma spatial transcriptomes (Extended Data Fig. 5a). Spatial projection validated CNVs that were identified in the recurrent but not in the primary meningioma from paired samples using targeted next generation DNA sequencing (Fig. 1b, 3c and Supplementary Table 2). Spatial projection also identified clonal CNVs from recurrent meningiomas in sub-clonal spatial transcriptomes from paired primary tumors that were below the limit of detection using bulk molecular approaches (Fig. 3c). In support of these data, spatial transcriptomes demonstrated decreased expression of MAPK genes (*RAB7, MAPK11, PLCE1*) or epigenetic regulators (*SMARCA2*) that were lost through copy number deletions in paired recurrent versus primary meningiomas (Fig. 3d). Interestingly, an intracranial meningioma overlying the frontal cortex (M8) also demonstrated divergent histological, SSV, CNV, and spatial transcriptomic architecture compared to patient-matched primary (M7) and recurrent (M7’) meningiomas overlying the occipital cortex (Fig. 1b, Extended Data Fig. 6a–i and Supplementary Table 1), suggesting regionally distinct meningiomas can be associated with divergent genomic features.

To determine if the diverse genomic mechanisms underlying high-grade meningioma recurrence were associated with convergent or divergent biochemical or cellular phenotypes, spatial protein profiling (Fig. 4a and Extended Data Fig. 7a–d) was performed alongside multiplexed sequential immunofluorescence (seqIF) to stain and image whole mount sections of primary (M4–7) and recurrent (M4’–7’) meningiomas (Fig. 4b, c, Extended Data Fig. 7e and Supplementary Table 7). Principal component analysis of spatial protein profiling data demonstrated divergent biochemical mechanisms in primary versus recurrent tumors (Extended Data Fig. 7a, b), but inspection of individual proteins revealed conserved trends underlying high-grade meningioma recurrence (Extended Data Fig. 7c). Proteins associated with cell proliferation (Ki-67) and PI3K-AKT signaling (PLCG1) were enriched in recurrent meningiomas, whereas proteins associated with MAPK signaling (pan-Ras), immune signaling (CD45, VISTA, CD14), and PI3K-AKT inhibition (INPP4B) were suppressed in recurrent meningiomas (Fig. 4a and Extended Data Fig. 7d). In support of these findings, multiplexed seqIF showed Ki-67 was enriched in recurrent versus primary meningioma cells that were marked by SSTR2A^46^ (Fig. 4c). Primary meningiomas were enriched in pan-Ras, INPP4B, macrophages (CD68, CD163) that were concentrated in the perivascular niche (CD31), and VISTA, an inhibitor of T cell activation (Fig. 4c). Meningiomas have poor responses to immune checkpoint inhibitors that target T cells^47,48^, and T cells marked by CD4 or CD8 were rare in either primary or recurrent meningiomas (Extended Data Fig. 7e). To validate these findings, meningioma cell types were deconvolved from spatial transcriptomes using single-cell RNA sequencing of 57,114 cells from 8 meningioma samples representing all DNA methylation groups^13^. Spatial deconvolution of single-cell types showed *CD163* macrophages, differentiated meningioma cells, *SSTR2A* meningioma cells, and non-cycling G1 phase meningioma cells were decreased in recurrent versus primary meningiomas (Fig. 4d). Cycling G2M phase and S phase meningioma cells were enriched in recurrent versus primary meningiomas (Fig. 4d).

To test the generalizability of mechanisms underlying meningioma recurrence revealed by spatial transcriptomic, protein profiling, multiplexed IF, and single-cell deconvolution approaches, MAPK and PI3K-AKT target gene expression was analyzed in primary (n = 403) versus recurrent meningiomas (n = 99) using bulk RNA sequencing data from independent meningiomas that were resected at UCSF (n = 200) or HKU (n = 302)^13,18^. In support of results from spatial approaches, MAPK target genes such as *DUSP1* (p = 0.0013) and *SPRY1* (p = 0.0059) were suppressed and PI3K-AKT target genes^49^ such as *SMC6* (p = 0.0011), *LSM4* (p = 0.0001), and *LARS* (p = 0.0007) were enriched in recurrent versus primary meningiomas (Student’s t tests) (Supplementary Table 8).

### Regionally distinct sub-clonal spatial transcriptomes, signaling mechanisms, and cell types underlie histological heterogeneity in high-grade meningiomas

High-grade meningiomas can arise *de novo*, progress from lower grade meningioma at the time of recurrence^50–52^, or may show predominantly low-grade histology with only focal evidence of high-grade treansformation^53^. Thus, regionally distinct histological or genomic intratumor heterogeneity can influence meningioma classification^8,19^, but the identity and spatial relationships among mechanisms driving intratumor heterogeneity in high-grade meningiomas are unknown. To address this limitation in our understanding of meningioma biology, spatial genomic and cellular mechanisms were studied across high-grade meningiomas demonstrating regionally distinct intratumor heterogeneity (M9–10) (Fig. 5a–b, Extended Data Fig. 8a-c and Supplementary Table 1).

Histological analyses of M9 revealed a well-demarcated area of increased cellularity, increased immunostaining for Ki-67, and increased mitotic count that was sufficient for diagnosis of WHO grade 3 meningioma in 1 of 2 regionally distinct cores (Fig. 5a). Both cores from M9 were otherwise comprised of WHO grade 2 histology, lower immunostaining for Ki-67, Hypermitotic meningioma DNA methylation grouping, and high gene expression risk scores but showed divergent SSVs inactivating epigenetic regulators (*ARID1A*, *ASXL1*) and divergent CNVs deleting chromosomes 4 and 14q that were only identified in the core with WHO grade 3 histology (Fig. 1b). Histological analyses of M10 revealed WHO grade 3 meningioma with mosaic immunostaining for p16 that inversely correlated with immunostaining for Ki-67 in 2 regionally distinct cores (Fig. 5b and Extended Data Fig. 8b). Both cores from M10 classified in the Hypermitotic meningioma DNA methylation group but showed divergent gene expression risk scores and divergent CNVs amplifying chromosome 1q or deleting chromosomes 4q, 9p, and 10q (Fig. 1b).

Spatial gene expression programs were analyzed across regionally distinct high-grade meningioma cores after sample integration and batch-correction with Harmony (Fig. 5c–f). Clusters C3, C6, and C9 in M9 correlated with WHO grade 3 histology (Fig. 5a, e) and differential expression analysis of spatial transcriptomes revealed shared enrichment of embryonic transcription factors (*SOX11*, *ELF3*) but divergent expression of meningeal homeostasis (*PTGDS* in C3) or immune genes (*CXCL8* in C6, *HLA-DPA1*, *IGHG1* in C9) in WHO grade 3 regions (Fig. 5g–i). Clusters C2, C8, and C10 in M9 correlated with WHO grade 2 histology that was immediately adjacent to the WHO grade 3 region and lacked embryonic transcription factor expression but was enriched in meningeal homeostasis (*PTGDS* in C2 and C10) or immune genes (*HLA-DPA1* in C8). Clusters C1, C4, C5, and C7 in M9 correlated with WHO grade 2 histology that was distant from the WHO grade 3 region and was enriched in tissue differentiation (*FIBIN* in C1 and C5, *ACTA2* in C4) and innate immune genes (*IFI27, IFIT3* in C7). M10 clusters C4, C5, and C6 correlated with reduced immunostaining for p16 (Fig. 5b, f), and differential expression analysis of spatial transcriptomes revealed shared enrichment of cell signaling and proliferation genes (*GPC1, CRABP1*) but divergent expression of immune genes in these regions (*IGHG1*, *IGKC*, *CLEC3B* in C6) (Fig. 5j–l). Cluster C8 correlated with intermediate immunostaining for p16 and demonstrated divergent cell signaling and proliferation genes (*MET*, *EGFL6*), supporting the hypothesis that regionally distinct mechanisms activating the cell cycle can exist in individual meningiomas (Fig. 2d, e). The remainder of M10 showed diffusely positive immunostaining for p16 and was enriched in senescence and cell cycle regulation genes (*MX2* in C7, *CDKN2B* in C9 and C10). Multiplexed seqIF showed that Ki-67 was enriched in the WHO grade 3 region of M9 and in the region of M10 with reduced immunostaining for p16 (Fig. 6a–c). Moreover, M9 and M10 showed regionally distinct expression of pan-Ras, INPP4B, CD68, CD163, VISTA, and the pericyte marker CD31. Spatial deconvolution of meningioma single-cell types^13^ validated regionally distinct changes in *CD163* macrophages, pericytes, endothelia, *SSTR2A* meningioma cells, extracellular matrix (ECM) remodeling meningioma cells, and G1/G2M/S phase meningioma cells in M9 and M10 (Fig. 6d). Thus, in support of the genomic, biochemical, and cellular phenotypes underlying temporal evolution of high-grade meningiomas (Fig. 3, 4 and Extended Data Fig. 4–7), regionally distinct cell proliferation, cell signaling, and immune mechanisms underlie spatial evolution of high-grade meningiomas.

## A preclinical platform for testing personalized medical therapies to overcome intratumor heterogeneity in high–grade meningiomas

Sub-clonal evolution underlies tumor recurrence and treatment resistance^1–5^, but preclinical models of intratumor heterogeneity or tumor evolution in response to treatment are scarce. To develop reagents to study high-grade meningioma heterogeneity and evolution in response to treatment, patient-derived high-grade M10G meningioma cells stably expressing CRISPRi machinery (M10G^dCas9 − KRAB^)^8,13^ were transduced with sgRNAs suppressing the cell cycle inhibitors *CDKN2A* (sg*CDKN2A*) or *CDKN2B* (sg*CDKN2B*), the epigenetic regulator *ARID1A* (sg*ARID1A*), or non-targeted control sgRNAs (sgNTC) (Extended Data Fig. 9a). RNA sequencing of triplicate M10G^dCas9 − KRAB^ cultures with differential expression and ontology analyses revealed *CDKN2A/B* suppression inhibited developmental and metabolic gene expression programs, whereas *ARID1A* suppression induced metabolic and mitotic gene expression programs (Fig. 7a and Supplementary Table 9). These data suggest drivers of high-grade meningioma intratumor heterogeneity, such as *CDKN2A/B* homozygous deletion or SSVs inactivating epigenetic regulators like *ARID1A* (Fig. 1b), may be associated with non-overlapping therapeutic vulnerabilities. In support of this hypothesis, preclinical experiments demonstrate meningiomas with loss of cell cycle regulators are susceptible to CDK4/6 inhibitors such as abemaciclib^13^, and meningiomas with loss of epigenetic regulators may be susceptible to histone deacetylase inhibitors such as vorinostat^17^.

To identify pharmacologic strategies inhibiting intratumor heterogeneity in high-grade meningiomas, M10G^dCas9 − KRAB^ cells transduced with sg*CDKN2A/B*, sg*ARID1A*, or sgNTC were reciprocally labeled with red or green fluorescence proteins to track pharmacologic responses and assembled into 3D organoid co-cultures for live cell microscopy. Abemaciclib blocked the growth of M10G^dCas9 − KRAB^ cells with *CDKN2A/B* suppression but did not block the growth of M10G^dCas9 − KRAB^ cells with *ARID1A* suppression or sgNTCs (Fig. 7b, c). To identify therapeutic vulnerabilities underlying meningiomas with loss of epigenetic regulators, spatial protein profiling was analyzed across 21 regions with or without SSVs inactivating *ARID1A* from M10 (Fig. 1e). These data revealed regionally distinct potential vulnerabilities to small molecule inhibitors of the DNA damage response (niraparib), EGFR signaling (erlotinib), MEK/ERK signaling (selumetinib), MET signaling (capmatinib), or PI3K-AKT signaling (copanlisib) (Fig. 7d). Vorinostat, niraparib, erlotinib, selumetinib, and copanlisib blocked the growth of M10G^dCas9 − KRAB^ cells expressing sgNTC, and selumetinib and copanlisib blocked the growth of cells with *ARID1A* suppression (Fig. 7e). To determine if combination molecular therapy could overcome intratumor heterogeneity in high-grade meningiomas, 3D organoid co-cultures of M10G^dCas9 − KRAB^ cells expressing sg*CDKN2A* and sgNTC (Fig. 7f), or sg*CDKN2A* and sg*ARID1A* (Fig. 7g), were treated with abemaciclib and selumetinib, or abemaciclib and copanlisib. Combination molecular therapy blocked the growth of meningioma cells with loss of *CDKN2A* and loss of *ARID1A* in both co-culture conditions and attenuated the growth of meningioma cells expressing sgNTC (Fig. 7h, i). Thus, high-grade meningiomas with loss of cell cycle and/or epigenetic regulators are susceptible to combination molecular therapy blocking CDK4/6, MEK/ERK signaling, and PI3K-AKT signaling.

## Discussion

Here we integrate spatial transcriptomics, spatial protein profiling, multiplexed seqIF, and spatial deconvolution of single-cell RNA sequencing across high-grade meningiomas to identify genomic, biochemical, and cellular mechanisms linking intratumor heterogeneity to the molecular, temporal, and spatial evolution of human cancer. Our results reveal divergent intratumor gene and protein expression programs distinguish high-grade meningiomas that are otherwise grouped together by the World Health Organization Classification of Central Nervous System Tumors^20^, one of the systems that is currently used to determine patient eligibility on clinical trials^6^. Analyses of matched pairs of primary and recurrent meningiomas reveal spatial expansion of sub-clonal copy number variants, decreased immune cell infiltration, decreased MAPK signaling, increased PI3K-AKT signaling, and increased cell proliferation underlie treatment resistance and tumor recurrence. We find regionally distinct high-grade meningioma samples displaying histological and molecular heterogeneity are associated with spatial gene expression programs that correlate with intratumor heterogeneity and cell proliferation. To translate these findings to clinical practice, we use epigenetic editing and lineage tracing approaches in human meningioma organoid models to identify new combinations of FDA-approved molecular therapies that target intratumor heterogeneity and block meningioma growth. In sum, our results establish a foundation for personalized medical therapy to treat patients with high-grade meningiomas and provide a framework for understanding mechanisms and therapeutic vulnerabilities driving intratumor heterogeneity and tumor evolution.

The human meningiomas in this study that were analyzed using bulk genomic, spatial transcriptomic, spatial protein profiling, multiplexed seqIF, and single-cell RNA sequencing deconvolution approaches were clinical FFPE samples, as opposed to fresh, frozen, or curated research specimens that are used for many exploratory investigations. Thus, the biological findings in this study may be generalizable to routine clinical practice. In support of this hypothesis, we show mechanisms underlying meningioma intratumor heterogeneity and evolution from our discovery cohort are conserved across a validation cohort comprised of 504 meningiomas from independent, international institutions.

Clinical trials of molecular therapy that are based on molecular inclusion criteria are underway for patients with meningiomas^6^. We identify divergent temporal evolution in recurrent versus primary meningiomas, suggesting that molecular analyses guiding clinical decision-making should be performed on recurrent tumor tissue rather than archival samples from prior resections. Our results also indicate that regionally distinct spatial evolution represents a barrier to accurate tumor classification and should be considered during histological or molecular analyses of meningiomas. Beyond classification, our preclinical model for testing personalized medical therapies to overcome intratumor heterogeneity may address the limitations molecular, temporal, or spatial evolution place on improving treatments for patients. Indeed, we show this system can enable medium-throughput screening of novel pharmacological strategies to treat tumors that are resistant to standard interventions. This system also suggests that meningioma cell growth patterns can be influenced by cell heterogeneity in the tumor microenvironment (Fig. 7b, f, g), and phenotypes such as these may hint at additional response or resistance mechanisms. To that end, clinical trials of abemaciclib (NCT02523014) or selumetinib (NCT03095248) as monotherapy for meningiomas are ongoing, but our data suggest that combination molecular therapy may be necessary to reverse the longstanding trend of non-positive clinical trials for patients with meningiomas^22,23,47,48^.

## Methods

### Inclusion and ethics

This study complied with all relevant ethical regulations and was approved by the UCSF Institutional Review Board (13–12587, 17–22324, 17–23196 and 18–24633). As part of routine clinical practice at UCSF, all patients who were included in this study signed a waiver of informed consent to contribute deidentified data to research projects.

### Meningiomas, clinical data, histology, and light microscopy

The study cohort consisted of 16 samples from 10 clinically aggressive meningiomas that were resected from 9 patients at UCSF from 2009 to 2021. Patient demographics, treatments, and clinical outcomes were recorded from the electronic medical record (Supplementary Table 1). Magnetic resonance imaging studies were reviewed to define meningioma locations and clinical outcomes. Detailed pathologic examination of the entire cohort was performed by a board-certified neuropathologist (C–H.G.L) to assess for histological or molecular heterogeneity. Histological and molecular grading were assigned using the 2021 WHO Classification of Central Nervous System Tumors^20^. For bulk sequencing analyses, meningioma tissue was isolated from formalin-fixed, paraffin-embedded (FFPE) blocks using biopsy punches (Integra Miltex Instruments, cat# 33–31-P/25). Genomic DNA was extracted from macro-dissected FFPE tumor tissue using the QIAamp DNA (Qiagen, cat# 56404) and the QIAamp RNeasy FFPE Tissue Kits (Qiagen, cat# 73504) at UCSF. For spatial profiling assays, 6 mm cores were punched from FFPE blocks using biopsy punches, and serial sections were mounted onto glass slides for spatial transcriptomic, protein profiling, H&E histology, or immunohistochemistry. Clinically validated immunohistochemistry for Ki-67 (DAKO, 1:50 dilution, MIB1 clone, cat# M7240), H3K27me3 (Cell Signaling, 1:50 dilution, C36B11 clone, cat# 9733, and p16 (MTM Labs, undiluted, E6H4 clone, cat# 9511) were performed at UCSF on core mounts with appropriate controls using a Leica Bond III platform and imaged using light microscopy on an BX43 microscope with standard objectives (Olympus). Images were obtained and analyzed using the Olympus cellSens Standard Imaging Software package (v1.16).

### DNA methylation profiling and analysis

Genomic DNA underwent bisulfite conversion using the EZ DNA Methylation kit (Zymo Research, cat# D5004), followed by amplification, fragmentation, and hybridization to Infinium EPIC 850k Human DNA Methylation BeadChips (Illumina, cat# 20020530) according to manufacturer’s instructions at the Molecular Genomics Core at the University of Southern California (Los Angeles, CA). Bioinformatic analysis was performed in R (v3.6.1). Meningioma DNA methylation data were preprocessed using the SeSAMe pipeline (Bioconductor v3.10) as previously described^13,54^. In brief, probes were filtered and analyzed using normal-exponential out-of-band background correction, nonlinear dye bias correction, p-value with out-of-band array hybridization masking, and β value calculation (β=methylated/[methylated+unmethylated]). Meningioma samples were assigned to Merlin-intact, Immune-enriched, or Hypermitotic DNA methylation groups using a support vector machine classifier, as previously described^13^.

### Targeted DNA sequencing and analysis

Targeted DNA sequencing was performed using the UCSF500 NGS panel, as previously described^26^. In brief, this capture-based next-generation DNA sequencing assay targets all coding exons of 479 cancer-related genes, select introns, and upstream regulatory regions of 47 genes to enable detection of structural variants such as gene fusions and DNA segments at regular intervals along each chromosome to enable genome-wide copy number and zygosity analyses, with a total sequencing footprint of 2.8 Mb (Supplementary Table 2). Multiplex library preparation was performed using the KAPA Hyper Prep Kit (Roche, cat# 07962355001). Hybrid capture of pooled libraries was performed using a custom oligonucleotide library (Nimblegen SeqCap EZ Choice). Captured libraries were sequenced as paired-end reads on an Illumina NovaSeq 6000 at >200x coverage for each sample. Sequence reads were mapped to the reference human genome build GRCh37 (hg19) using the Burrows-Wheeler aligner (v0.7.17). Recalibration and deduplication of reads was performed using the Genome Analysis Toolkit (v4.3.0.0). Coverage and sequencing statistics were determined using Picard (v2.27.5) CalculateHsMetrics and CollectInsertSizeMetrics. Single nucleotide variant and small insertion/deletion mutation calling was performed with FreeBayes, Unified Genotyper, and Pindel. Large insertion/deletion and structural alteration calling was performed with Delly. Variant annotation was performed with Annovar. Single nucleotide variants, insertions/deletions, and structural variants were visualized and verified using Integrative Genome Viewer (v.2.16.0). Genome-wide copy number and zygosity analysis was performed by CNVkit and visualized using NxClinical (Biodiscovery, v6.0).

### Targeted RNA sequencing and analysis

Targeted gene expression profiling was performed using a hybridization and barcode-based RNA sequencing NanoString panel, with quality control from internal negative and spike-in positive controls on the NanoString nCounter Analysis System at the San Francisco Veterans Affairs Core (San Francisco, CA). 200 ng of total RNA per sample was hybridized to barcoded reporter probes and biotin-conjugated capture probes from a custom codeset targeting genes of interest at 65C for 16 hours according to manufacturer instruction. Hybridization mixtures were washed and target/probe complexes were purified and bound to streptavidin coated cartridges. Cartridges were scanned on the nCounter Digital Analyzer with a FOV setting of 550. Gene expression risk scores spanning 0 to 1, with a greater value denoting higher risk of recurrence, were calculated using a previously trained and validated algorithm based on Lasso Cox regression and bootstrap aggregation using log2-transformed, housekeeping gene normalized gene expression counts from a 34-gene signature as input. Previously identified cutoffs were used (low risk ≤0.3761, high risk >0.5652)^24^.

### Spatial transcriptome sequencing and analysis

Spatial transcriptomic profiling was performed on FFPE sections using the 10x Genomics Visium Spatial assay (v1, cat# 1000336). 6 mm cores were mounted within capture areas on Visium glass slides, deparaffinized, stained with H&E, and imaged at the Gladstone Institutes Histology Core (San Francisco, CA). Libraries were prepared according to manufacturer instructions at the Gladstone Institutes Genomics Core (San Francisco, CA). Libraries were sequenced on an Illumina NovaSeq 6000 instrument at the UCSF Center for Advanced Technology. Sequencing was performed with the recommended protocol (read 1: 28 cycles, i7 index read: 10 cycles, i5 index read: 10 cycles, and read 2: 91 cycles). FASTQ sequencing files and histology images were processed using the 10x SpaceRanger pipeline and the Visium Human Transcriptome Probe Set v1.0 GRCh38–2020-A. Data were visualized using the 10x Loupe Browser software (v6.3.0). Principal component analysis (PCA) was run on the normalized filtered feature-barcode matrix to reduce the number of feature (e.g. gene) dimensions. Uniform manifold approximation and projection (UMAP) analysis was used to visualize spatial transcriptomes in a 2D space. Graph-based clustering was performed to cluster spatial transcriptomes with related expression profiles together based on their concordance in PCA space. Differential expression analyses were performed using mean gene expression in each cluster, log2 fold-change of gene mean expression in a cluster relative to all other spatial transcriptomes, and a p-value denoting gene expression significance in each cluster relative to spatial transcriptomes in other clusters. P-values in each cluster were adjusted for false discovery rate to account for the number of genes being tested. Heatmaps of spatial transcriptomic data were generated in the Loupe Browser, which considers the top N genes for each cluster, sorted by log2 fold-change (by default N = 120/X, where X is the total number of spatial transcriptome clusters). Heatmaps were generated using hierarchical clustering with euclidean distance and average linkage.

Spaceranger generated filtered feature matrices were imported into a Seurat object (v4.3.0, arguments min.cells=3, min.features=100) using R (v4.2.1) and RStudio (v2022.07.2 Build 576) (Supplementary Table 4). The individual count matrices were normalized by nFeature_RNA count (subset=nFeature_RNA>1500 and nFeature_RNA<9500) and integrated with Harmony (v0.1.1). Optimal cluster resolution was determined using Clustree (v0.5.0, analyzing resolutions 5, 2, 1, 0.9, 0.8, 0.7, 0.6, 0.6, 0.5, 0.4, 0.3, 0.1, 0.0), and subsequent principal component (npcs=30) and UMAP (dims=1:30, min.dist=0.2) analyses were performed. UMAP projections and cluster distributions were visualized in the Loupe browser after combining spatial transcriptomic data from individual capture areas using the 10x Spaceranger aggr pipeline (v2.0.0). CNV analysis from spatial transcriptomes was performed using inferCNV (v1.14.0) and spatialinferCNV (v0.1.0). Capture areas of interest were combined with an additional capture area containing a geographic population of non-neoplastic cells, using the 10x Spaceranger aggr pipeline and Harmony, as described above. The cluster distribution was visually assessed in the Loupe browser to identify the cluster containing non-neoplastic tissue such as brain or endothelial. All cluster annotations were exported into a csv file and imported into R, along with the aggregate filtered feature matrix. The count matrix, annotated clusters, and a gene order file were input into inferCNV (arguments: cutoff=0.1, cluster_by_groups=TRUE, HMM = TRUE, denoise=TRUE) to generate a six-state CNV probability model for each spatial transcriptomic cluster. Deconvolution of meningioma cell types from single-cell RNA sequencing was performed using SCDC (v 0.0.0.9000). To do so, each spatial transcriptome was treated as a pseudobulked RNA sequencing dataset and leveraged against known cell types from a reference single-cell RNA sequencing dataset comprised of 57,114 cells from 8 human meningioma samples representing all DNA methylation groups^13^. Spatial and single-cell transcriptomic data were separately processed for quality control using QC filtering, normalization, dimensionality reduction, and clustering. Single-cell transcriptomic data were subsampled to 1000 cells per cell type, and the top differentially expressed genes were selected for each cell type. Using this expression set, spatial transcriptomes were deconvolved to yield a matrix with predicted proportions of cell type for each spatial transcriptome, which were visualized using SpatialFeatureplot (Seurat v3).

### Spatial protein profiling and analysis

Spatial protein profiling was performed on FFPE sections using the NanoString Digital Spatial Profiler at the UCSF Laboratory for Cell Analysis Genome Core (San Francisco, CA). Meningioma sections were labeled with DAPI and a multiplexed cocktail of 78 oligo-conjugated antibodies (Supplementary Table 5) using human protein panel modules generated at NanoString Technologies (Seattle, WA). H&E stained whole slide images were overlayed on fluorescent DAPI projections and 200μm regions of interest were annotated based on histological and morphological heterogeneity by a board-certified neuropathologist (C–H.G.L). Oligonucleotides were released from regions of interest using ultraviolet cleavage, aspirated tags were hybridized to optical barcodes, and processed using the NanoString nCounter Analysis System. Barcodes were first normalized with internal spike-in controls and then normalized against housekeeping genes. Principal components analysis was performed using the prcomp function in R (v3.6.1) using default settings.

### Multiplexed sequential immunofluorescence (seqIF) and microscopy

Automated multiplexed seqIF staining and imaging was performed on FFPE sections at Northwestern University using the COMET platform (Lunaphore Technologies). The multiplexed panel was comprised of 29 antibodies (Supplementary Table 7). The 29-plex protocol was generated using the COMET Control Software, and reagents were loaded onto the COME device to perform seqIF. All antibodies were validated using conventional IHC and/or IF staining in conjunction with corresponding fluorophores and 4’,6-diamidino-2-pheynlindole counterstain (DAPI, ThermoFisher Scientific, cat# 62248). For optimal concentration and best signal-to-noise ratio, all antibodies were tested at 3 different dilutions, starting with the manufacturer-recommended dilution (MRD), MRD/2, and MRD/4. Secondary Alexa fluorophore 555 (ThermoFisher Scientific, cat# A32727) and Alexa fluorophore 647 (ThermoFisher Scientific, cat# A32733) were used at 1/200 or 1/400 dilutions, respectively. The optimizations and full runs of the multiplexed panel were executed using the seqIF technology integrated in the Lunaphore COMET platform (characterization 2 and 3 protocols, and seqIF protocols, respectively). The seqIF workflow was parallelized on a maximum of 4 slides, with automated cycles of iterative staining of 2 antibodies at a time, followed by imaging, and elution of the primary and secondary antibodies, with no sample manipulation during the entire workflow. All reagents were diluted in Multistaining Buffer (Lunaphore Technologies, cat# BU06). The elution step lasted *2*min for each cycle and was performed with Elution Buffer (Lunaphore Technologies, cat# BU07-L) at 37°C. Quenching lasted for 30sec and was performed with Quenching Buffer (Lunaphore Technologies, cat# BU08-L). Imaging was performed with Imaging Buffer (Lunaphore Technologies, cat# BU09) with exposure times set at 4min for all primary antibodies, except P16 antibody at 8min, and secondary antibodies at 2min. Imaging was performed with an integrated epifluorescent microscope at 20x magnification. Image registration was performed immediately after concluding the staining and imaging procedures by COMET Control Software. Each seqIF protocol resulted in a multi-stack OME-TIFF file where the imaging outputs from each cycle were stitched and aligned. COMET OME-TIFF files contain a DAPI image, intrinsic tissue autofluorescence in TRITC and Cy5 channels, and a single fluorescent layer per marker. Markers were subsequently pseudocolored for visualization of multiplexed antibodies.

### Cell culture and molecular biology

M10G cells^8^ were cultured in a medium comprised of Advanced DMEM/F12 (Gibco, cat# 12634) supplemented with 5% FBS, B-27 supplement without vitamin A (Gibco, cat #12587010), N-2 supplement (Gibco, cat# 17502048), 100U/ml Anti-anti (Gibco, cat# 15240), 1% CTSTMGlutaMAXTM-1 (Gibco, cat# A1286001), 20ng/ml EGF (R&D Systems, cat# 236EG200), and 20ng/ml FGF basic/FGF2 (R&D Systems, cat# PRD23350). HEK293T cells (ATCC, cat# CRL-3216) were cultured in Advanced DMEM (Gibco, cat# 12491015) supplemented with 3% FBS and CTSTMGlutaMAXTM-1. Lentiviral particles containing pMH0001 (UCOE-SFFV-dCas9-BFP-KRAB, Addgene, cat# 85969) were produced by transfecting HEK293T cells with standard packaging vectors using the TransIT-Lenti Transfection Reagent (Mirus, cat# 6605). M10G cells were transduced with lentiviral particles to generate M10G^dCas9-KRAB^ cells. Successfully transduced cells were isolated through selection of BFP positive cells using fluorescence activated cell sorting on a Sony SH800. Single-guide RNA (sgRNA) protospacer sequences suppressing *CDKN2A*, *CDKN2B*, or *ARID1A* were individually ligated into the pCRISPRia-v2 vector83 (Addgene, cat# 84832) between the BstXI and BlpI sites. Each vector was verified by Sanger sequencing of the protospacer. Lentivirus was generated as described above for each sgRNA expression vector. M10G^dCas9-KRAB^ cells were transduced with lentivirus from each sgRNA expression vector and selected to purity using 20μg/mL puromycin over 7 days.

### Cell culture quantitative reverse-transcriptase polymerase chain reaction

RNA was extracted from M10G cells using RNeasy Plus Mini Kit (Qiagen, cat# 74134) and cDNA was synthesized using the iScript cDNA Synthesis Kit (Bio-Rad, cat# 1708891). Target genes were amplified using PowerUp SYBR Green Master Mix and QuantStudio 6 thermocycler (Thermo Fisher Scientific). Gene expression was calculated using the DDCt method, with normalization to *GAPDH* (sense: 5’-ATGGGGAAGGTGAAGGTCG-3’, antisense: 5’-GGGGTCATTGATGGCAACAATA-3’). Target gene primers included *CDKN2A* (sense: 5’-ATGGAGCCTTCGGCTGACT-3’, antisense: 5’-GTAACTATTCGGTGCGTTGGG-3’), *CDKN2B* (sense: 5’-ACGGAGTCAACCGTTTCGGGAG-3’, antisense: 5’-GGTCGGGTGAGAGTGGCAGG-3’), and *ARID1A* (sense: 5’-CCTGAAGAACTCGAACGGGAA-3’, antisense: 5’-TCCGCCATGTTGTTGGTGG-3’).

### Cell culture RNA sequencing and analysis

RNA was extracted from triplicate M10G cultures (sgNTC, sgCDKN2A, sgCDKN2B, sgARID1A) using the RNeasy Plus Mini Kit (Qiagen, cat#74134). 1ug of RNA from each condition was shipped to Medgenome (Foster City, CA) for bulk RNA sequencing (Supplementary Table 9). Quality control was performed using FASTQC (v0.11.9) and the results were aggregated using MultiQC (v1.12). Adapter sequences and bases with quality scores <30 at the 3’ and 5’ ends of the reads were trimmed using Cutadapt (v3.7). Trimmed treads that were less than 20 bases in length were discarded. Processed reads were mapped to the reference genome GRCh38 using HISAT2 (v2.2.0) with default parameters. FeatureCounts (v2.0.0) was used to extract gene expression counts. The resulting count matrix was used to perform differential gene expression analysis with DESeq2 (v1.36.0).

Gene Set Enrichment Analysis (GSEA, v4.3.2) was performed to determine whether differentially expressed in M10G cultures belonged to common biological pathways. Gene rank scores were calculated using the formula: sign(log_2_ fold-change) × −log10(p-value). Pathways were defined using the gene set file Human_GOBP_AllPathways_no_GO_iea_December_01_2022_symbol.gmt, which is maintained by the Bader laboratory. Gene set size was limited to range between 15 and 500, and positive and negative enrichment files were generated using 2000 permutations. The EnrichmentMap App (v3.3.4) in Cytoscape (v3.7.2) was used to visualize the results of pathway analysis. Nodes with FDR q value < 0.05 and p-value < 0.05, and nodes sharing gene overlaps with Jaccard + Overlap Combined (JOC) threshold of 0.375 were connected by blue lines (edges) to generate network maps. Clusters of related pathways were identified and annotated using the AutoAnnotate app (v1.3.5) in Cytoscape that uses a Markov Cluster algorithm to connect pathways by shared keywords in the description of each pathway. The resulting groups of pathways were designated as the consensus pathways in a circle.

### Meningioma organoid pharmacology and microscopy

CRISPRi-modified and fluorescently-labeled M10G^dCas9-KRAB^ meningioma cells for 3D organoid experiments were generated by mixing sgNTC-mScarlet with sg*CDKN2A*-FumGW cells, sgNTC-GFP with sg*ARID1A*-mCherry cells, or sg*CDKN2A*-FumGW with sg*ARID1A*-mCherry cells 1:1. For pharmacologic experiments, a minimum of 2000 cells were seeded into each well of a PrimeSurface ultra-low attachment V-shaped 96 well plate (S-Bio, cat# MS-9096V). The following day, meningioma organoids were transferred to a spheroid microplate (Corning, cat# 4515) prior to beginning 12 days of continuous drug treatment. Organoids were maintained in a medium comprised of Advanced DMEM/F12 (Gibco, cat# 12634) supplemented with B-27 supplement without vitamin A (Gibco, cat# 12587010), N-2 supplement (Gibco, cat# 17502048), 100U/ml Anti-anti (Gibco, cat# 15240), 1% CTSTMGlutaMAXTM-1 (Gibco, cat# A1286001), 20ng/ml EGF (R&D Systems, cat# 236EG200), and 20ng/ml FGF basic/FGF2 (R&D Systems, cat# PRD23350). A Zeiss Cell Observer Spinning Disc Confocal microscope fitted with a temperature and carbon dioxide-controlled chamber was used to acquire fluorescence images of live meningioma organoids during drug treatments using Plan-Apochromat 10x/1.3 air objective.

### Statistics

All experiments were performed with independent biological replicates and repeated, and statistics were derived from biological replicates. Biological replicates are indicated in each figure panel or figure legend. No statistical methods were used to predetermine sample sizes, but sample sizes in this study are similar or larger to those reported in previous publications. Data distribution was assumed to be normal, but this was not formally tested. Investigators were blinded to conditions during clinical data collection and analysis of mechanistic or functional studies. Bioinformatic analyses were performed blind to clinical features, outcomes or molecular characteristics. The clinical samples used in this study were retrospective and nonrandomized with no intervention, and all samples were interrogated equally. Thus, controlling for covariates among clinical samples is not relevant. Cells and animals were randomized to experimental conditions. No clinical, molecular, or cellular data points were excluded from the analyses. Unless specified otherwise, lines represent means, and error bars represent standard error of the means. Results were compared using Student’s *t*-tests, which are indicated in figure legends alongside approaches used to adjust for multiple comparisons. In general, statistical significance is shown by asterisks (*p£0.05, **p£0.01, ***p£0.0001), but exact p-values are provided in the figure legends when possible.

### Reporting summary

Further information on research design is available in the Nature Research Reporting Summary linked to this article.

## Data Availability

DNA sequencing and spatial transcriptomic data that support the findings of this study have been deposited in the Sequence Read Archive (https://www.ncbi.nlm.nih.gov/sra) under BioProject ID PRJNA950017. DNA methylation, and RNA sequencing data that support the findings of this study have been deposited in the NCBI Gene Expression Omnibus (https://www.ncbi.nlm.nih.gov/geo/) under accession numbers GSE228316 (DNA methylation) and GSE228433 (RNA sequencing). The publicly available GRCh38 (hg38) and CRCh37.p13 (hg19) were used in this study. Source data are provided with this paper.
